# Does creatine cause hair loss? A 12-week randomized controlled trial

**DOI:** 10.1080/15502783.2025.2495229

**Published:** 2025-04-23

**Authors:** Mohammadyasin Lak, Scott C. Forbes, Damoon Ashtary-Larky, Sahar Dadkhahfar, Reza Mahmoud Robati, Farshid Nezakati, Makan Khajevandi, Sara Naseri, Arvin Gerafiani, Neda Haghighat, Jose Antonio, Grant M. Tinsley

**Affiliations:** a Department of Exercise Physiology, Sport Sciences Research Institute of Iran, Tehran, Iran; b Department of Physical Education Studies, Brandon University, Brandon, MB, Canada; c Nutrition and Metabolic Diseases Research Center, Ahvaz Jundishapur University of Medical Sciences, Ahvaz, Iran; d Skin Research Center, Shahid Beheshti University of Medical Sciences, Tehran, Iran; e South Tehran Branch, Payame Noor University, Tehran, Iran; f Department of Sport Physiology, Tehran Central Branch, Islamic Azad University, Tehran, Iran; g Department of Sport Physiology, Karaj Branch, Islamic Azad University, Karaj, Iran; h Karaj Branch, Islamic Azad University, Karaj, Iran; i Laparoscopy Research Center, Shiraz University of Medical Sciences, Shiraz, Iran; j Department of Health and Human Performance, Nova Southeastern University, Davie, FL, USA; k Department of Kinesiology & Sport Management, Texas Tech University, Lubbock, TX, USA

**Keywords:** Testosterone, dihydrotestosterone, alopecia, creatine monohydrate

## Abstract

**Background:**

Creatine is a widely used ergogenic aid that enhances muscle strength and lean mass. However, concerns have been raised about the potential role in promoting hair loss by increasing dihydrotestosterone (DHT). Currently, there is no direct evidence examining the relationship between creatine supplementation and hair follicle health. Therefore, the purpose was to determine the effects of 12 weeks of creatine supplementation on androgen levels and hair follicle health in healthy young males.

**Methods:**

Forty-five resistance-trained males (ages 18–40 years) were recruited and randomly assigned to either a creatine monohydrate (5 g/day) or placebo (5 g maltodextrin/day) group. Participants maintained their habitual diets and training routines. Blood samples were collected at baseline and after 12 weeks to measure total testosterone, free testosterone, and DHT. Hair follicle health was assessed using the Trichogram test and the FotoFinder system (hair density, follicular unit count, and cumulative hair thickness). Statistical analyses were performed using repeated measures ANOVA, and potential outliers were examined through sensitivity analysis.

**Results:**

Thirty-eight participants completed the study, with no significant differences in baseline characteristics between groups. There were no group-by-time interactions observed for any hormones or hair-related outcomes (*p* > 0.05). While total testosterone increased (∆ = post value minus pre value: creatine = ∆124   ±   149 ng/dL; placebo = ∆216   ±   203 ng/dL) and free testosterone decreased (creatine = ∆-9.0   ±   8.7 pg/mL; placebo = ∆-9   ±   6.4 pg/mL) over time, these effects were independent of supplementation. There were no significant differences in DHT levels, DHT-to-testosterone ratio, or hair growth parameters between the creatine and placebo groups.

**Conclusion:**

This study was the first to directly assess hair follicle health following creatine supplementation, providing strong evidence against the claim that creatine contributes to hair loss.

## 1. Introduction

Creatine, a ubiquitous non-protein amino acid, is among the most extensively researched and commonly utilized ergogenic aids in both professional and recreational athletes [1–4]. Creatine supplementation combined with resistance training augments gains in muscle strength and lean tissue mass [1,5–7]. Mechanistically, creatine supplementation elevates skeletal muscle phosphocreatine (PCr) and free creatine stores, facilitating rapid re-synthesis of adenosine triphosphate (ATP) during exercise. In addition, creatine influences cell swelling, satellite cell activation and differentiation, hormonal responses (e.g. insulin-like-growth factor-1), myogenic regulatory factors, protein kinetics, inflammation, and oxidative stress, which may further improve muscle performance and recovery [8]. Despite these established benefits, claims that creatine leads to hair loss through an increase in androgen hormones have persisted [3].

Testosterone and its more bioactive metabolite, dihydrotestosterone (DHT), may play crucial roles in muscle hypertrophy and androgenic effects [9]. DHT is synthesized from testosterone via the enzyme 5-alpha reductase and has been shown to exhibit a stronger binding affinity to androgen receptors compared to testosterone [10]. In 2009, a study conducted on male young athletes found that short-term (3-week) creatine supplementation enhanced the testosterone-to-DHT conversion, potentially increasing serum DHT [11]. Given the well-documented role of DHT in male pattern baldness (androgenetic alopecia) [12], concerns have emerged regarding whether creatine supplementation could accelerate hair loss in predisposed individuals. Additionally, some athletes have anecdotally reported experiencing hair loss while using creatine. However, these reports are based on subjective self-observations, and no controlled studies have systematically examined the potential relationship between creatine supplementation and hair loss.

To date, only one short-term study on male athletes has reported increased DHT levels [11]; however, these findings remain inconsistent, and no subsequent research has either confirmed or refuted these results [13,14]. It is important to note that no mechanistic studies have directly linked creatine supplementation to hair follicle physiology or androgen-driven hair loss. Moreover, the long-term impact of creatine on DHT levels and hair loss remains largely unknown. Therefore, we performed a randomized controlled trial aimed to address a significant gap in the literature by evaluating, for the first time, the effects of creatine supplementation on androgenic hormone modulation and its potential role in hair loss by examining changes in testosterone, DHT, and hair follicle health in resistance-trained individuals undergoing 12 weeks of creatine supplementation.

## 2. Methods

### 2.1. Participants

Forty-five resistance-trained male participants were recruited for this study. Recruitment was conducted via an online advertisement designed to identify individuals meeting the study’s eligibility criteria. Eligible participants were required to be biological males between the ages of 18 and 40 years and have at least one year of consistent and structured resistance training experience. Participants with a history of hair transplantation or medical procedures related to hair restoration were excluded, as were those with prior use of medications commonly prescribed for the prevention or treatment of hair loss, including finasteride or minoxidil. The use of dietary supplements specifically aimed at promoting hair growth or creatine supplementation within the three months prior to enrollment also disqualified participants from the study. Individuals with diagnosed endocrine disorders that could potentially affect androgen metabolism were also not eligible to participate. Additional exclusion criteria included the presence of dermatological or systemic conditions known to influence hair loss, such as alopecia areata or thyroid disorders. Participants who had used anabolic-androgenic steroids or other hormonal enhancement agents within the preceding six months were excluded. Furthermore, individuals with a history of scalp disorders that could interfere with trichological assessments, an inability to commit to the 12-week intervention period, or failure to adhere to study protocols – such as consistent supplement intake or attendance at scheduled assessments – were not eligible to participate. All participants provided informed consent and agreed to maintain their usual dietary habits and resistance training routines throughout the 12-week intervention period.

### 2.2. Study protocol

Participants were instructed to maintain their habitual diet throughout the intervention period to ensure a stable caloric intake. They were also required to engage in a minimum of three resistance training sessions per week during the 12-week intervention period. Weekly check-in reports were used to monitor compliance with training.

### 2.3. Randomization and blinding

Participants were randomly assigned to one of two groups, either the creatine monohydrate group or the placebo group, using a computerized randomization process in a double-blind design. The randomization sequence was generated by an independent researcher who was not involved in data collection or analysis to ensure unbiased allocation. To maintain allocation concealment, sequentially numbered opaque sealed envelopes were used for assigning participants to their respective groups. At the start of the study, twenty participants were allocated to the creatine monohydrate group, while nineteen were assigned to the placebo group. Blinding was implemented at two levels: 1. Participant blinding: Participants were unaware of their group assignment, as both the creatine monohydrate and placebo supplements were provided in identical, unlabeled powder form; 2. Investigator blinding: The researchers responsible for data collection and analysis remained blinded to group allocation until the conclusion of the study and data analysis to minimize potential bias.

### 2.4. Participant retention and dropouts

**Figure 1.**
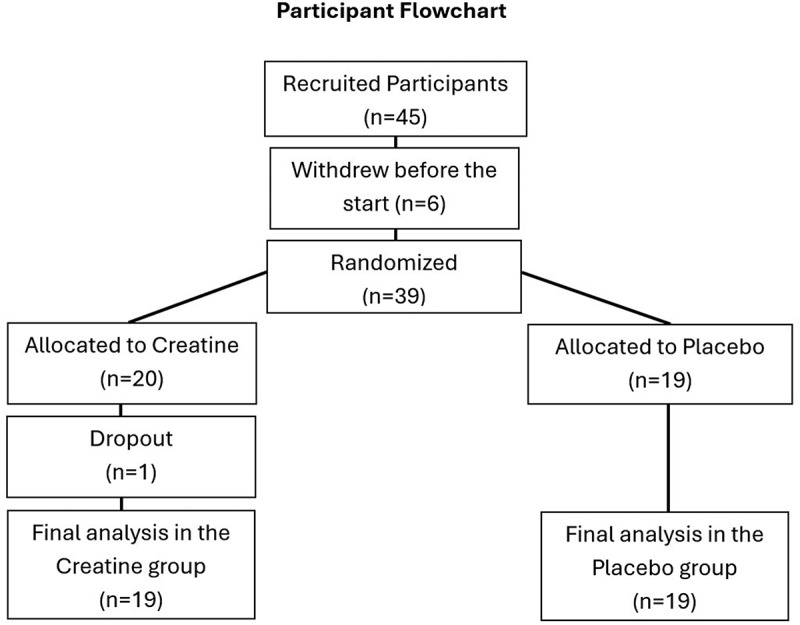
Flowchart of participant enrollment, randomization, and analysis.

Initially, six participants withdrew from the study prior to the commencement of the intervention due to personal reasons. During the study, one participant from the creatine group dropped out for a personal reason unrelated to the study. As a result, 19 participants remained in each group for the final analysis. Data from the participant who withdrew was excluded from the final analysis. Additionally, one participant from the placebo group did not complete the final hair assessment, and only blood-related data were included for this individual. Figure 1 shows the flow of participants through the study, including randomization, withdrawals, and final analysis.

### 2.5. Ethical considerations

The study was conducted in collaboration with and under the supervision of the Iran Skin Research Center to ensure compliance with clinical and methodological standards. The study was approved by the Ethics Committee of Shiraz University of Medical Sciences (approved code: IR.SUMS.SCHEANUT.REC.1403.021). Furthermore, this trial was registered in the Iranian Registry of Clinical Trials (IRCT) under the registration number IRCT20240814062762N1.

### 2.6. Supplementation protocol

Participants were supplemented daily with either creatine monohydrate or a placebo over a 12-week intervention period. The creatine group received 5 g of creatine monohydrate (BPI Sports Nutrition, USA) per day, while the placebo group was provided with an equivalent dose of 5 g of maltodextrin as a non-active control. Both substances were supplied in powdered form and were identical in appearance, texture, and taste to maintain the integrity of the double-blind study design.

Participants were instructed to dissolve their assigned powder in water and consume it at the same time each day to ensure consistency. Adherence to the supplementation was monitored daily through WhatsApp check-ins conducted by a research assistant who was not involved in data collection and analysis. Participants were required to send confirmation messages indicating the completion of their daily supplement intake. Compliance was further verified through the collection of empty supplement packets at scheduled bi-weekly follow-up visits. To minimize variability, they were instructed to maintain their habitual diets and adhere to their regular resistance training regimens throughout the intervention period.

### 2.7. Assessment methods

#### 2.7.1. Blood biomarker assessments

Blood samples were collected at baseline (Day 1) and at the end of the 12-week intervention to evaluate relevant biomarkers. The measured biomarkers included estimated glomerular filtration rate (eGFR), creatinine, total testosterone, free testosterone, and dihydrotestosterone (DHT). To minimize inter-sample variability, all blood samples were drawn from the antecubital vein at a consistent time of day for all participants. Participants were not required to be in a fasted state during sampling. Immediately after collection, the blood samples were processed to preserve integrity. Serum separation was performed using centrifugation at 3000 rpm. The separated serum was then stored at  − 70°C until analysis. All laboratory tests were conducted according to standardized procedures, and each sample was analyzed in triplicate to enhance accuracy and reliability.

#### 2.7.2. Hair assessment

Hair growth and loss parameters were evaluated using the Trichogram test and the FotoFinder system (FotoFinder Systems GmbH, Germany). The hair assessments were conducted by board-certified dermatologists at the Iran Skin Research Center to ensure high accuracy and reliability in the evaluation process.

To ensure accurate and reproducible results, a series of standardized pre-evaluation conditions were implemented. Participants were instructed to avoid washing their hair for at least 24 to 48 hours before the assessment to preserve the natural sebum layer on the scalp, which is essential for accurate imaging and analysis. The use of hair styling products, including gels, sprays, mousses, creams, and medicated shampoos, was prohibited to prevent interference with the imaging process or alterations in evaluation outcomes. Additionally, participants were required to refrain from hair coloring, bleaching, or chemical treatments (e.g. keratin treatments, rebonding) for at least two weeks before the test, as these procedures could modify hair structure and potentially compromise the accuracy of the assessment.

To ensure hair remained in its natural growth phase, participants were instructed not to cut or trim their hair for at least three weeks before the evaluation. This restriction was particularly important for the Trichogram test, which requires an analysis of hair in different stages of the growth cycle. Furthermore, participants were advised to avoid excessive alcohol and caffeine consumption for at least 24 hours before the assessment, as these substances may induce temporary physiological changes in the scalp and hair, potentially affecting evaluation results.

The hair assessments were conducted under controlled conditions, with standardized lighting and magnification settings to reduce variability. Imaging was focused primarily on the vertex region of the scalp, which is a critical area for evaluating hair density, growth patterns, and hair follicle health. The vertex is particularly significant because it is one of the most common sites for progressive hair thinning and androgenetic alopecia. By selecting this region, the assessment provided a more reliable analysis of hair loss progression, miniaturization patterns, and overall follicular health.

**Figure 2.**
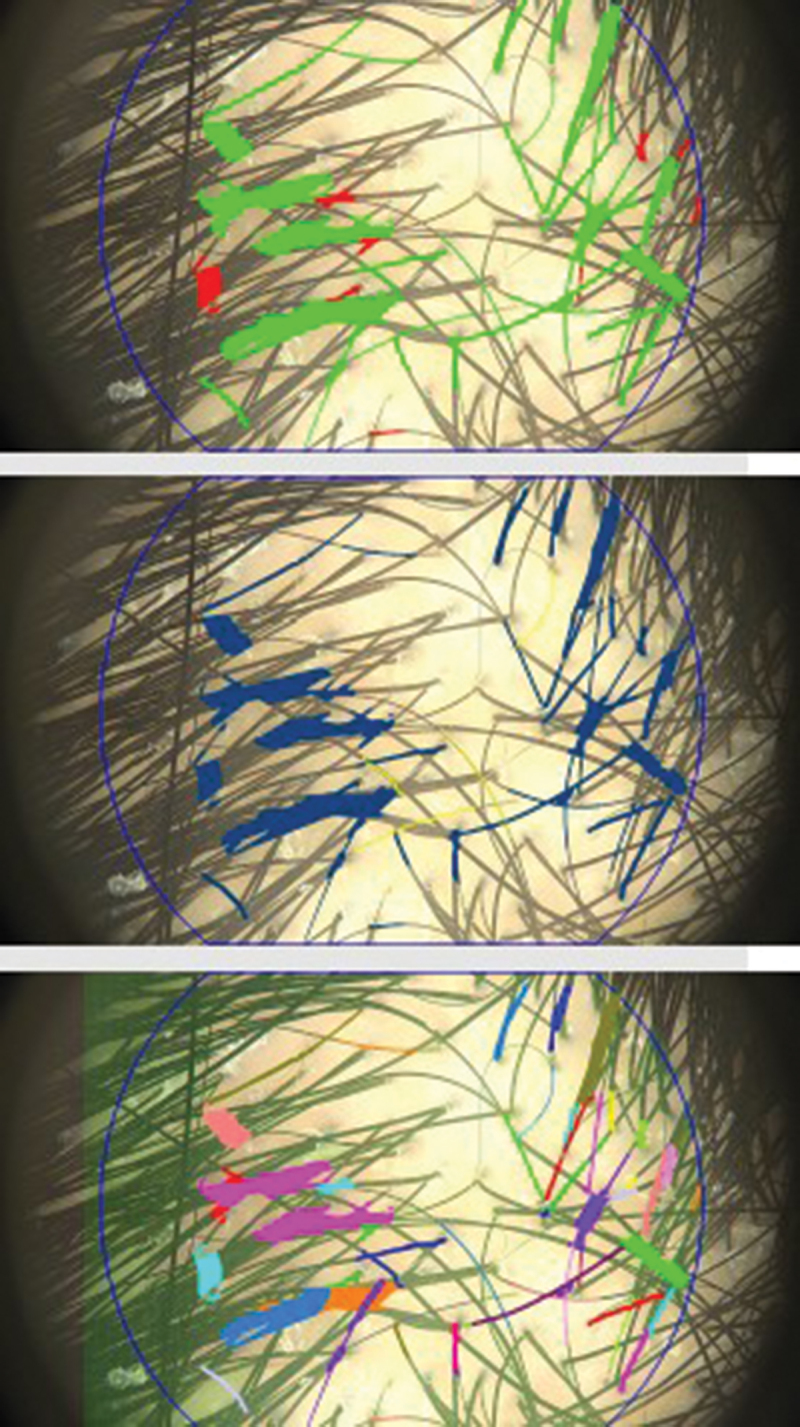
Representative Trichogram test images from the study.   The assessments were conducted using the FotoFinder system under standardized conditions to analyze hair shaft thickness, follicular density, and overall hair health.

Using the FotoFinder system, high-resolution images of the vertex were captured under uniform lighting conditions, ensuring minimal variability in assessment results. A representative image from the Trichogram test is provided in Figure 2 to illustrate the hair assessment process. The system’s polarized light technology and dermoscopic magnification allowed for a detailed evaluation of hair shaft thickness, follicular density, and potential scalp abnormalities. These standardized imaging conditions enabled the detection of subtle changes in hair characteristics over time, enhancing the reliability of the assessments. By focusing on the vertex, the study aimed to provide a clinically relevant and reproducible evaluation of hair growth and loss patterns, making it an essential parameter in assessing treatment efficacy and progression of hair disorders.

## 3. Statistical analysis

Initially, independent samples t-tests were performed to determine if there were any significant differences between groups were present at baseline (Table 1). Across outcome variables, no significant differences were observed (p: 0.31 to 0.99). Subsequently, repeated measures analysis of variance (RMANOVA) was used to assess the effects of time (pre vs. post; within-subjects factor) and group (Creatine vs. Placebo; between-subjects factor) on each outcome variable using the *afex* R package (v. 1.4–1). Generalized eta-squared effect sizes, which represent the proportion of variance in the outcome variable attributable to the specific term (i.e. group, time, or group × time) were generated to accompany each RMANOVA term. Estimated marginal means with within-subject error bars, along with raw individual data points, are presented to visualize the data. Using the *performance* R package (v. 0.12.0), homogeneity of variances was evaluated using Levene’s test, and normality of residuals was assessed using QQ plots: two minor deviations of homogeneity of variance (0.04 < *p* < 0.05) were observed for testosterone and cumulative hair thickness; QQ plots indicated approximate normality for most variables. Due to these potential assumption violations, additional rank-based RMANOVA tests were performed to determine whether the primary analysis results were influenced by these deviations. As indicated in the Results, the major findings were practically identical in the original and rank-based analyses. To account for multiple outcome variables and terms, the false discovery rate correction was employed. Statistical significance was accepted at *p* < 0.05 for corrected p-values. Analysis was performed in R (v. 4.4.2) by a blinded study investigator, with unblinding taking place after finalizing the analysis.

**Table 1.** Baseline characteristics.

	All (n = 38)		Creatine Group (n = 19)		Placebo Group (n = 19)		
	Mean	SD	Mean	SD	Mean	SD	*p*
Age (y)	26.7	5.6	26.0	4.6	27.3	6.5	0.48
Body mass (kg)	82.6	10.6	81.8	11.2	83.5	10.2	0.63
Height (cm)	180.0	5.3	179.6	5.5	180.3	5.3	0.70
BMI (kg/m^2^)	25.5	3.2	25.3	3.2	25.7	3.2	0.73
Testosterone (ng/dL)	298.3	76.1	288.0	76.0	308.5	76.9	0.42
Free Testosterone (pg/mL)	21.8	7.6	21.9	8.5	21.7	6.7	0.94
DHT (ng/dL)	52.9	20.7	52.8	16.9	52.9	24.4	0.99

*p* from independent samples t-test. Abbreviations: BMI – body mass index; DHT – dihydrotestosterone.

In the analysis of raw data, each combination of time and group was examined for the presence of extreme outliers (i.e. values above Q3  +  3 × IQR or below Q1–3 × IQR) using the rstatix R package (v. 0.7.2). The following extreme outliers were identified in the raw data: Creatine group, Pre – free testosterone (1); Creatine group, Post – none; Placebo group, Pre – hair rate terminal (1), hair rate vellus (1), DHT (2), and DHT: T (2); and Placebo group, Post – cumulative hair thickness (2) and DHT: free T (1). For each of these outcomes, sensitivity analysis was performed by conducting the main statistical analysis (i.e. RMANOVA on raw data) with and without these outliers. No material differences, including general magnitude of effects and statistical significance, were observed in the results of the sensitivity analysis. As such, the analysis from the full dataset is included in the main text, with the sensitivity analysis presented in the supplemental materials. Additionally, as described, rank-based tests that are not susceptible to influence by outliers were performed in the full dataset due to possible violations of assumptions.

## 4. Results

**Figure 3.**
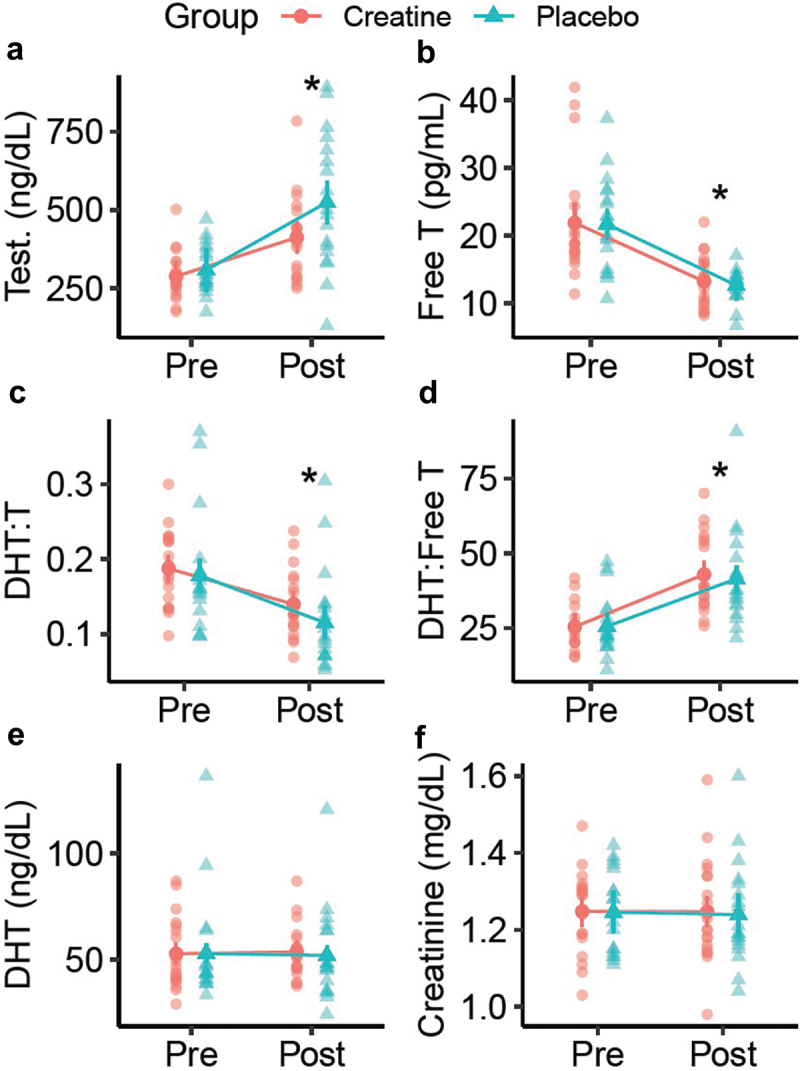
Hormonal changes. Raw changes in total testosterone (a), free testosterone (b), dihydrotestosterone (DHT)-to-testosterone ratio (c), DHT-to-free testosterone ratio with equivalent units (d), DHT (e), and creatinine (f) between pre and post time points are presented. Data points represent individual values and estimated marginal means. Error bars correspond to within-subjects error bars based on the experimental design. *Indicates a statistically significant main effect of time from repeated measures analysis of variance. No group main effects or group x time interactions were observed.

**Figure 4.**
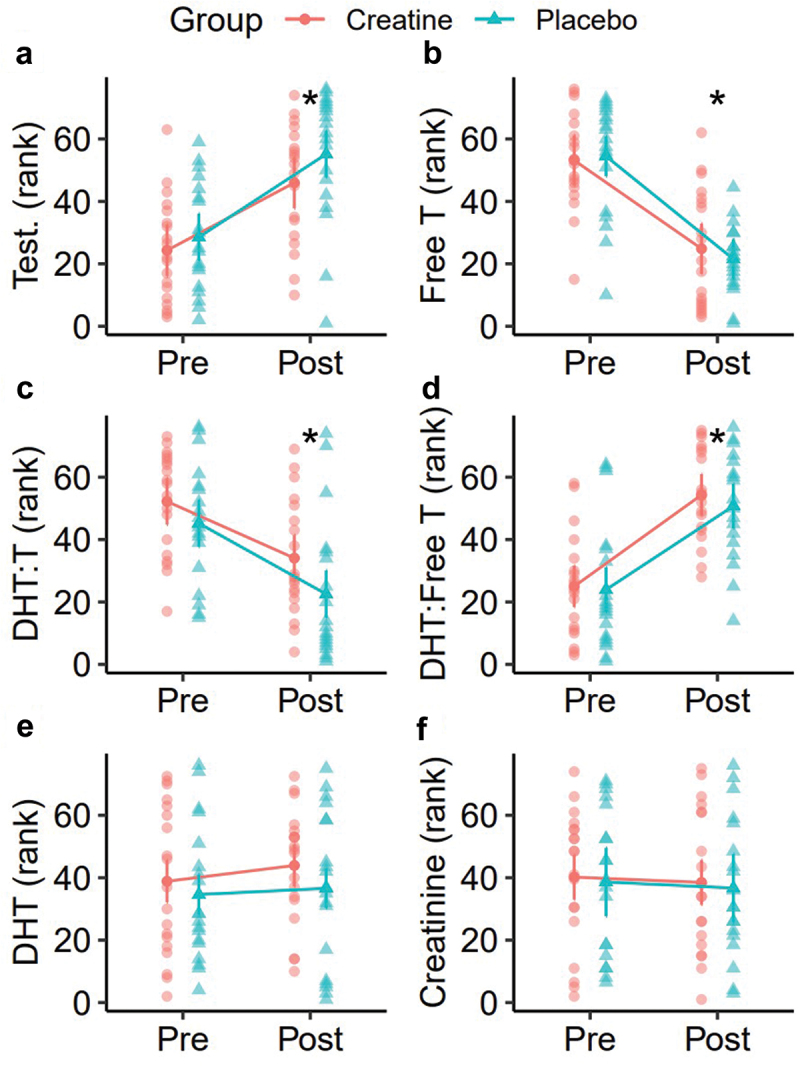
Hormonal changes (ranks). Rank-based changes in total testosterone (a), free testosterone (b), dihydrotestosterone (DHT)-to-testosterone ratio (c), DHT-to-free testosterone with equivalent units (d), DHT (e), and creatinine (f) between pre and post time points are presented. Data points represent individual values and estimated marginal means. Error bars correspond to within-subjects error bars based on the experimental design. *Indicates a statistically significant main effect of time in rank-based repeated measures analysis of variance. No group main effects or group x time interactions were observed.

**Figure 5.**
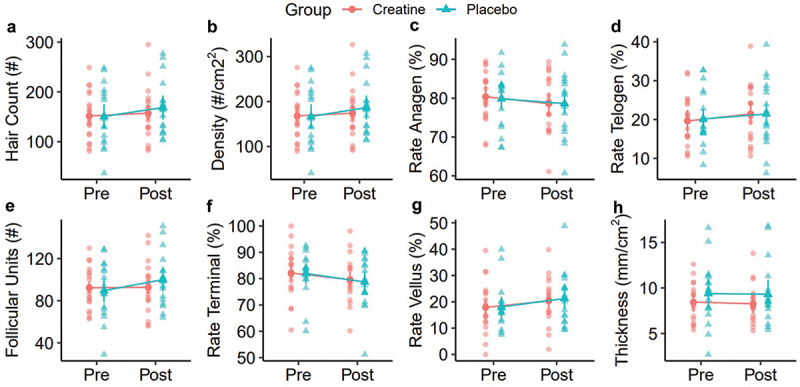
Hair outcomes. Raw TrichoScale trichogram results are presented before and after the supplementation period. Outcomes included the hair count (a; total number of hairs counted in the analyzed area), hair density (b; total number of hairs per square centimeter), hair rate anagen (c; percentage of hairs in the anagen [growth] phase), hair rate telogen (d; percentage of hairs in the telogen [resting] phase), total follicular units (e; total number of hair groupings in the analyzed area), hair rate terminal (f; percentage of terminal [thicker, longer, mature] hairs), hair rate vellus (g; percentage of vellus [fine, short, soft] hairs), and cumulative thickness (h; total thickness of all hairs in the analyzed area). Data points represent individual values and estimated marginal means. Error bars correspond to within-subjects error bars based on the experimental design. No main effects or group x time interactions were observed in the repeated measures analysis of variance.

**Figure 6.**
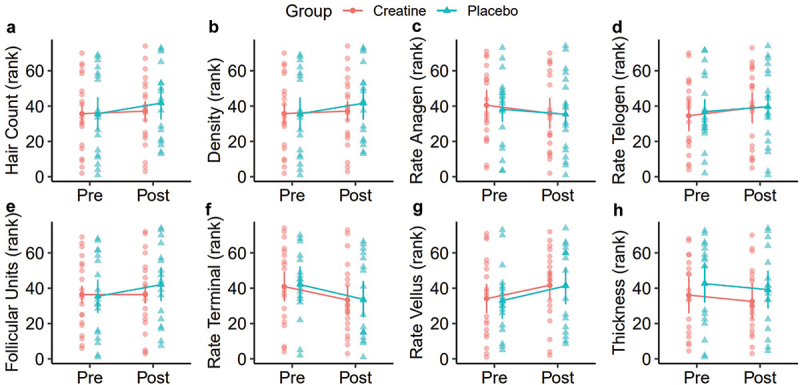
Hair outcomes (ranks). Rank-based TrichoScale trichogram results are presented before and after the supplementation period. Outcomes included the hair count (a; total number of hairs counted in the analyzed area), hair density (b; total number of hairs per square centimeter), hair rate anagen (c; percentage of hairs in the anagen [growth] phase), hair rate telogen (d; percentage of hairs in the telogen [resting] phase), total follicular units (e; total number of hair groupings in the analyzed area), hair rate terminal (f; percentage of terminal [thicker, longer, mature] hairs), hair rate vellus (g; percentage of vellus [fine, short, soft] hairs), and cumulative thickness (h; total thickness of all hairs in the analyzed area). Data points represent individual values and estimated marginal means. Error bars correspond to within-subjects error bars based on the experimental design. No main effects or group x time interactions were observed in the rank-based repeated measures analysis of variance tests.

Thirty-eight participants completed the present study (Table 1). However, one participant was missing hair assessments and was excluded from the analysis of hair outcomes. In the analysis of raw data, rank-based analysis, and sensitivity analysis (Table 2, Supplemental Table S1), no group × time interactions were observed for any outcome. In all versions of the analysis, statistically significant time main effects were observed for testosterone (T), free T, the dihydrotestosterone (DHT): T ratio, and the DHT: free T ratio (Figures 3 and 4, Supplemental Figure S1). These effects indicated increased total testosterone and DHT:free T over time, as well as decreased free T and DHT: T, regardless of group (*p* < 0.001 for each). No changes in raw DHT concentrations, creatinine, or eGFR were observed. There were no reported side effects in either group over the duration of the study. Across all hair outcomes, no statistically significant effects of group, time, or group × time were observed (Table 2). This finding was consistent across all versions of the analysis (Figures 5 and 6, Supplemental Figure S2).

**Table 2.** RMANOVA results.

	Analysis of Raw Data							Rank-based Analysis					
Variable	Term	Df	MSE	F	p	ges	p (FDR)	Df	MSE	F	p	ges	p (FDR)
eGFR (mL/min)	Group	36	553.47	0.05	0.83	0.001	0.95	36	876.70	0.01	0.91	0.000	0.97
	Time	36	65.25	0.04	0.84	0.000	0.95	36	132.35	0.36	0.55	0.001	0.97
	Group* Time	36	65.25	0.04	0.84	0.000	0.95	36	132.35	1.36	0.25	0.005	0.92
Creatinine (mg/dL)	Group	36	0.02	0.02	0.88	0.000	0.95	36	643.15	0.08	0.77	0.001	0.97
	Time	36	0.01	0.03	0.87	0.000	0.95	36	368.12	0.17	0.68	0.002	0.97
	Group* Time	36	0.01	0.01	0.91	0.000	0.95	36	368.12	0.00	0.98	0.000	0.98
Testosterone (ng/dL)	Group	36	20402.11	4.08	0.05	0.060	0.46	36	415.19	2.06	0.16	0.034	0.90
	Time	36	15847.87	34.57	**0.00**	0.296	<0.0001*	36	266.41	41.53	0.00	0.311	<0.0001*
	Group* Time	36	15847.87	2.51	0.12	0.030	0.69	36	266.41	0.44	0.51	0.005	0.97
Free Testosterone (pg/mL)	Group	36	40.07	0.07	0.79	0.001	0.95	36	289.85	0.07	0.80	0.001	0.97
	Time	36	29.30	50.70	**0.00**	0.373	<0.0001*	36	227.37	78.41	0.00	0.489	<0.0001*
	Group* Time	36	29.30	0.03	0.87	0.000	0.95	36	227.37	0.45	0.51	0.005	0.97
DHT (ng/dL)	Group	36	652.86	0.02	0.90	0.000	0.95	36	805.37	0.79	0.38	0.018	0.95
	Time	36	99.81	0.00	0.98	0.000	0.98	36	184.90	1.28	0.27	0.007	0.92
	Group* Time	36	99.81	0.15	0.70	0.001	0.95	36	184.90	0.24	0.63	0.001	0.97
DHT: T	Group	36	0.01	0.94	0.34	0.020	0.95	36	506.32	3.22	0.08	0.057	0.52
	Time	36	0.00	33.00	**0.00**	0.176	<0.0001*	36	241.56	32.80	0.00	0.227	<0.0001*
	Group* Time	36	0.00	0.61	0.44	0.004	0.95	36	241.56	0.40	0.53	0.004	0.97
DHT: Free T	Group	36	195.28	0.04	0.84	0.001	0.95	36	393.00	0.26	0.61	0.005	0.97
	Time	36	89.36	59.35	**0.00**	0.341	<0.0001*	36	203.96	73.31	0.00	0.410	<0.0001*
	Group* Time	36	89.36	0.14	0.71	0.001	0.95	36	203.96	0.15	0.70	0.001	0.97
Hair Count (#)	Group	35	4116.84	0.12	0.73	0.003	0.95	35	716.46	0.13	0.72	0.003	0.97
	Time	35	1611.51	1.55	0.22	0.012	0.70	35	235.18	1.11	0.30	0.008	0.92
	Group* Time	35	1611.51	0.43	0.51	0.003	0.95	35	235.18	0.41	0.52	0.003	0.97
Hair Density (#/cm2)	Group	35	5041.51	0.12	0.73	0.002	0.95	35	715.92	0.12	0.73	0.003	0.97
	Time	35	1978.12	1.51	0.23	0.012	0.70	35	236.24	1.06	0.31	0.007	0.92
	Group* Time	35	1978.12	0.45	0.51	0.004	0.95	35	236.24	0.41	0.53	0.003	0.97
Hair Rate Anagen (%)	Group	35	80.85	0.02	0.90	0.000	0.95	35	694.10	0.05	0.82	0.001	0.97
	Time	35	29.14	1.48	0.23	0.011	0.70	35	261.62	0.96	0.33	0.007	0.92
	Group* Time	35	29.14	0.05	0.82	0.000	0.95	35	261.62	0.05	0.82	0.000	0.97
Hair Rate Telogen (%)	Group	35	80.85	0.02	0.90	0.000	0.95	35	694.10	0.05	0.82	0.001	0.97
	Time	35	29.14	1.48	0.23	0.011	0.70	35	261.62	0.96	0.33	0.007	0.92
	Group* Time	35	29.14	0.05	0.82	0.000	0.95	35	261.62	0.05	0.82	0.000	0.97
Total Follicular Units (#)	Group	35	820.45	0.12	0.73	0.002	0.95	35	728.98	0.14	0.71	0.003	0.97
	Time	35	304.53	1.81	0.19	0.014	0.70	35	221.15	0.91	0.35	0.006	0.92
	Group* Time	35	304.53	1.51	0.23	0.012	0.70	35	221.15	0.91	0.35	0.006	0.92
Hair Rate Terminal (%)	Group	35	122.16	0.04	0.84	0.001	0.95	35	579.79	0.02	0.90	0.000	0.97
	Time	35	49.39	3.09	0.09	0.025	0.56	35	351.14	3.31	0.08	0.034	0.52
	Group* Time	35	49.39	0.04	0.84	0.000	0.95	35	351.14	0.01	0.93	0.000	0.97
Hair Rate Vellus (%)	Group	35	122.16	0.04	0.84	0.001	0.95	35	579.79	0.02	0.90	0.000	0.97
	Time	35	49.39	3.09	0.09	0.025	0.56	35	351.14	3.31	0.08	0.034	0.52
	Group* Time	35	49.39	0.04	0.84	0.000	0.95	35	351.14	0.01	0.93	0.000	0.97
Cumulative Thickness (mm/cm2)	Group	35	7.90	2.29	0.14	0.033	0.70	35	486.52	1.64	0.21	0.024	0.92
	Time	35	7.21	0.05	0.83	0.001	0.95	35	448.19	0.50	0.48	0.007	0.97
	Group* Time	35	7.21	0.01	0.94	0.000	0.96	35	448.19	0.00	0.98	0.000	0.98

Abbreviations: RMANOVA – repeated measures analysis of variance; Df – Denominator degrees of freedom; MSE – Mean square error; F – F value; ges – generalized eta-squared effect size; FDR – false discovery rate; eGFR – estimated glomerular filtration rate; DHT – Dihydrotestosterone; T – Testosterone.

## 5. Discussion

Creatine has been well-established as a dietary supplement to enhance resistance training adaptations including gains in muscular strength and lean tissue mass. Despite these beneficial effects, there is a persistent fear among some consumers that creatine may increase the conversion of testosterone to DHT, which has been linked to male pattern baldness. To date, a single study found an increase conversion of DHT in male athletes and no study has directly examined hair follicle metrics. The major finding of the current study was that, in a randomized double-blind placebo-controlled trial, there was no effect of 12 weeks of supplementation compared to placebo on changes in DHT or testosterone. Further, there were no changes over time or between groups for any of the hair outcomes. In addition, there were no changes in creatinine (often used as a surrogate marker of kidney dysfunction) and eGFR. These findings provide evidence to refute the claim that creatine causes hair loss.

The speculation that creatine causes hair loss appears to come from a single paper that used a randomized placebo-controlled crossover design in male rugby players (*N* = 20), whereby particpants supplemented with creatine (25 g/day with 25 g/day of glucose for 7 days followed by 5 g/day of creatine with 25 g/day of glucose for 14 days) compared to placebo [11]. There was no significant change in serum testosterone levels over time, however DHT increased 56% following the loading phase and remained 40% above baseline during the maintenance phase. The placebo condition had a slight decrease (although not significant) over time from 1.26  ±  0.52 to 1.09  ±  0.40 nmol/L after the loading phase to 1.06  ±  0.43 nmol/L after the maintenance phase, while the creatine condition increased over time from 0.98  ±  0.37 to 1.53  ±  0.5 nmol/L after the loading phase and to 1.38  ±  0.45 nmol/L following the maintenance phase. Importantly, all these values at all time points in both conditions were within normal physiological ranges (healthy adult males aged 18–59 years: 0.8–3.5 nmol/L). Further, the ratio of DHT:T was elevated by 36% after the loading phase and remained 22% higher than baseline during the maintenance phase. Previous research comparing androgen levels with pre-mature male pattern baldness revealed that the DHT: T ratio was elevated in plasma and indirectly confirms the high activity of the 5 alpha-reductase type 2 [15]. Testosterone, the primary circulating androgen in males, can be metabolized to DHT via 5 alpha-reductase type 2, furthermore, based on the strong affinity for DHT for androgen receptors, DHT has been implicated in the pathogenesis of alopecia [16]. Specifically, DHT can bind to androgen receptors in susceptible hair follicles and cause them to shrink, leading to hair loss. However, our current results contradict previous findings showing an increased conversion of testosterone to DHT, as we found no significant differences between conditions for DHT or DHT:T ratio. These differences may be related to methodological differences. For example, the current study did not use a loading phase and was longer in duration (12 weeks vs. 3 weeks). In addition, and in support of the lack of differences in androgen levels, we did not find any differences in any hair outcome (hair count, density, hair rate anagen, hair rate telogen, total follicular units, hair rate terminal, hair rate vellus, and cumulative thickness). To our knowledge, this is the first study to directly assess hair follicle quality and quantity following creatine supplementation. Interestingly, we did find significant main effects of time for testosterone (increase) and free testosterone (decrease). Speculatively, these findings may be associated with the time of year but do not appear to be influenced by creatine supplementation. The assessments were conducted in late summer and in the first month of autumn. Previous research has reported seasonal variations in testosterone levels [17], however, based on the current research design we are unclear why there was a main effect of time and future research may be warranted. To date, there is some, albeit limited, evidence demonstrating a small increase in testosterone follow creatine supplementation [18], however, the majority of studies found no increase in testosterone [13,14,19–23]. Overall, based on our current study and the majority of the evidence, creatine does not influence testosterone (free or total) and DHT.

There are several limitations to the current study that warrant caution. First, this study was only conducted in males. Males were selected because DHT has been linked to male pattern baldness, however, future research may be needed in females. The study was only 12 weeks in duration, it is possible that a longer term and higher dose creatine study may alter androgens and hair. However, the selected dose is a commonly ingested dose of creatine, and we found no trend or evidence of any changes in hair outcomes. Further, only plasma samples were taken to assess androgen levels, future research may consider directly assessing hair androgen levels. Lastly, family history of hair loss was not determined. Males with biological fathers with hair loss were twice as likely to have hair loss than males with fathers experiencing no hair loss [24], highlighting the importance of monitoring family history in future research.

In summary, creatine supplementation appears to be a safe supplement. There were no changes in creatinine or eGFR following 12 weeks of creatine supplementation (5 g/day) in healthy young resistance trained males. Further, there were no significant differences between groups for changes in testosterone, DHT, or DHT:T ratio. Lastly, there were no changes over time or between groups for any hair outcomes. These results refute the common claim that creatine causes baldness.
